# A randomized trial of over‐the‐counter hearing aids for adults with cognitive decline due to Alzheimer’s Disease and related dementias

**DOI:** 10.1002/alz70861_108922

**Published:** 2025-12-23

**Authors:** Kimberly D Mueller, Victoria J. Williams, Nathaniel A. Chin, Pamela E. Souza

**Affiliations:** ^1^ Wisconsin Alzheimer's Disease Research Center, University of Wisconsin‐Madison, School of Medicine and Public Health, Madison, WI USA; ^2^ Department of Communication Sciences and Disorders, University of Wisconsin‐Madison, Madison, WI USA; ^3^ Wisconsin Alzheimer’s Institute, University of Wisconsin‐Madison School of Medicine and Public Health, Madison, WI USA; ^4^ University of Wisconsin‐Madison, Madison, WI USA; ^5^ Department of Medicine, University of Wisconsin‐Madison School of Medicine and Public Health, Madison, WI USA; ^6^ Alzheimer's Disease Research Center, University of Wisconsin‐Madison School of Medicine and Public Health, Madison, WI USA; ^7^ Northwestern University, Evanston, IL USA

## Abstract

**Background:**

The 2024 Lancet Commission highlights hearing loss as a leading modifiable risk factor for Alzheimer’s and related dementia (ADRD). Despite its prevalence in aging, and its even higher occurrence in individuals ADRD, hearing loss often remains unaddressed. Contributing factors include communication challenges being misattributed to cognitive decline rather than hearing loss. Both patients and providers may also lack awareness of accessible treatments such as over‐the‐counter (OTC) hearing aids. This presentation describes baseline data from a randomized clinical trial (RCT) investigating benefits of OTC hearing aids offered to participants at time of diagnosis of either MCI or early ADRD.

**Method:**

Hearing tests are embedded within the diagnostic flow of the memory appointment and conducted by a staff member trained to use a tablet audiometer. Results are reviewed by an off‐site audiologist and patients receive information about their hearing and recommendations for onward care, regardless of eligibility for the trial. At baseline, trial participants complete the Hearing Handicap Inventory for the Elderly (HHIE) and study partners complete the Perception of Conversation Index‐Dementia of the Alzheimer’s Type (PCI‐DAT).

**Result:**

Approximately 15% of patients diagnosed with ADRD were hearing aid users. 30% who received hearing tests did not qualify for the study because they had hearing loss outside the fitting range for OTC hearing aids. Patients enrolled in the study to date (80% male, mean age 77 yrs) demonstrated mild loss through 2 kHz sloping to a moderate high‐frequency loss bilaterally. Despite mild loss, all participants self‐reported significant unaided hearing handicap. We also examined communication ability as rated by primary communication partners, who were spouses (80% of the cohort), adult children, or close friends. In contrast to significant hearing handicap ratings by participants, partners rated communication as not problematic; the mean score on PCI‐DAT (Subscale 1) was 39 of a maximum possible score of 154, where higher scores represent more frequent and more problematic conversation difficulties.

**Conclusion:**

This report offers a snapshot of hearing status, unaided communication ability, and patient views on hearing care in a group of community‐dwelling older adults with MCI or early ADRD.

